# Extracts and Gleanings

**Published:** 1871-02

**Authors:** 


### PART II.

### Abridged Extracts and Gleanings from our Exchanges

*MULTUM IN PARVO.*

RETRACTED NIPPLE.—Dr. Geo. H. Lyman, Boston, Mass. (Boston Med. and Surg. Journal,) at a late meeting of the Boston Obstetrical Society, described the manner in which the child's tongue "strips" the nipple between its tongue and upper jaw, as a milker strips the cow's udder with his fingers. He had observed the operation in the mouth of an infant with hare lip.

He had also related the case of a woman whose nipple was so poorly developed as to be apparently on a level with the breast. After confinement the breast could not be evacuated; the consequence of which was an excessively troublesome abscess. In her next pregnancy the plan was adopted of breaking off the neck of an ordinary wine bottle (with smooth lips), and binding it on to the breast in such a manner that the circular rim of glass pressed upon the areola around the base of the nipple. This was done for ten days preceding confinement, and the result was most satisfactory. Not only was a deep circular depression made around the nipple, but the latter became more elevated; and the success of the experiment was established by the ease with which the child, when born, accomplished the act of sucking.—*Cincinnati Lancet and Observer*, September, 1870.

ITCH TEATED BY BALSAM OF PERU.—Balsam of Peru is claimed to be an effectual remedy for itch. The treatment is as follows: The patient is stripped and well and carefully rubbed over with Peru balsam, from crown to heel, avoiding the production of abrasions. It soaks into the galleries and eggs, and kills everything on the body within an hour. A second rubbing, ten days after, destroys any stray animalculæ or products of eggs that may by chance have been in the clothes at the first rubbing, and thus does away with any need for baking or otherwise disinfecting the clothing.—*Edin. Mid. Jour.* May, 1870.—*American Jour. Med. Sci.*—*Nashville Journal of Medicine and Surgery*, Sept. 1870.

SULPHITE OF SODA IN TINEA CAPITIS, ETC.—In the American Jour. of Medical Science for October, 1869, Dr. Charles M. Watrous, of Pennsylvania, recommends the Sulphite of Soda, as a local application in tinea capitis, crusta lacta and similar affections. In one case he used half an ounce to a pint of water, applied constantly by compresses wet with the solution—making it weaker as it caused smarting. In another case he used forty grs. of the sulphite to half an ounce each of distilled water and glycerine, the parts moistened with this three or four times a day. This last prescription he has used in the ear three times a day in scrofulous otitis, dropping in a few drops after washing the ear out, and excluding the air by cotton wool.—*Oregon Med. and Surgical Reporter.*

Flatulent Distension of the Colon with or without Diarrhea.—We take the following short extract from a paper by Dr. Habershon, of Guy's Hospital, on functional disease of the colon ?

To check the flatulent distension of the colon from gaseous evolution, creosote and carbolic acid hold a deservedly prominent position. And when there is also diarrhea, carbolate of lime may be given internally with marked benefit. The carbolate of lime as prepared by Mr. Squire, of Oxford street, is perfectly white, and the smell is not so strong as the ordinary carbolate of lime used as a disinfecting agent. I have generally given it in one grain doses combined with henbane; and I may remark that in the diarrhœa of phthisis, where there is evidence of fermentative changes in the colon, I have repeatedly used the carbolate of lime with good effect.—*Oregon Med. and Surgical Reporter*, Apr. 1870.

Treatment of Consumption.—Dr. Goodwin W. Timms, one of the physicians to the North London Consumption Hospital (Medical and Surgical Reporter, from the Dublin Medical Press and Circular), concludes an article on this subject as follows :

We may sum up shortly our treatment of consumption : Light, simple and nourishing diet, in quantity always in proportion to the appetite of the individual, (the palate is an excellent guide, which we should always be afraid to offend), all wholesome fruits and vegetables, a moderate amount of thoroughly cooked meat, and diluents, tea, milk, whey, &c., according to the patient's experience, avoiding all stimulants and forcing of the appetite. Regimen : excitement of the skin by constant cleanliness, friction and woolen clothing ; fresh air—sea air, if possible ; exercise of every kind, gymnastic exercises, singing, reading ; the avoidance of every restriction by dress upon the chestwalls and of indolence and self-indulgence of every kind ; the exclusion of gas from all apartments inhabited by the invalid ; early hours, and as short a sojourn as possible in the same atmosphere; hence it is better to take a short sleep in the day than to remain more than six or seven hours in the bed-room, the windows of which should never be shut except on particular occasions, or under peculiar circumstances.

Drug treatment. An obstinate cough, with expectoration, in the member of a consumptive family, unaccompanied by much general disturbance, is most successfully treated by twelve or fifteen drops of dilute hydrochloric acid in one ounce of water every two hours. Patients often declare that they taste the chlorine in the expectoration.—*Medical Archives.*—*American Eclectic Medical Review*, June, 1870.

Opium in Diabetes.—According to Dr. Pavy, opium, morphia and codeine, all possess the property of checking the elimination of sugar by the urine. Codeine is most powerful, commencing with one half-grain dose and going on to ten-grain doses thrice a day. A man, aged fifty, after restricted diet and alkalies, was passing urine of specific gravity 10.38, with 26.64 grains of sugar per ounce. After 30 days of opium there was no sugar, and the specific gravity of the urine was 10.23. On leaving off the opium again, the specific gravity rose to 10.48, and 10.65 grains of sugar were passed in 24 hours. On renewing the opium, the sugar immediately lessened, although he continued to take ordinary food. A woman, aged 68, passed five pints of urine of 10.40 specific gravity, and sugar to the extent of 32.72 grains daily. By the use of opium, with ordinary diet, the urine in three months was of specific gravity 19.16, and quite free from sugar. She continued well for twelve months; then anxiety and overwork renewed the disease. Again three months of codeine completely cured her.—*Dublin Medical Press and Circular. Boston Medical and Surgical Journal*, June 1870.

Chloroform—Pro et Con.—Under this heading, Prof. N. R. Smith, of Baltimore, expresses the opinion in the Baltimore Medical Journal, that while in surgical manipulations in which incisions and loss of blood are not involved, and hence no danger of pyæmia, as in the reduction of dislocations and in operations on the eye, for instance, which require the patient to be completely passive and non-resistant, and in the case of nervous and timid persons who would refuse surgical operations, but for the confidence they place in chloroform, the use of the anæsthetic is highly advantageous, he asserts confidently, as the experience of half a century, that pyæmia or inchorrhemia and septicemia after operations under chloroform, are more common than when it has not been employed. This he attributes both to the more frequent secondary hemorrhage, and to the morbid influence of the anæsthetic on the functions of the nervous system, and its influence upon the constitution of the blood.—*St. Louis Medical Archives.*

Large Doses of Opium in Tetanus.—In view of the increased attention which probably will be given to the use of choral in the treatment of tetanus by reason of the encouraging results already obtained, it should not be forgotten that heroic doses of opium have also proved in many instances successful. *L' Union Medicale* gives the experience of Dr. Chazarin, who practiced for seven years in the French colony of Senegal. He mentions altogether twenty-eight cases, twenty of which, treated by various means, terminated in death. The eight others were submitted to the following treatment: First day, 15 grains of gummy extract of opium in solution; second day 22 grains; third day, 30 grs.; fourth day, 37 grains; fifth day, 45 grains; and so on, increasing the dose each day seven grains if the symptoms did not improve. When 90 grains were reached, the doses were diminished in the same ratio from day to day. Of these eight patients only one died, and this in consequence of frictions of oil of turpentine imprudently undertaken on the advice of a neighbor. These cases deserve particular attention, though they are not very uncommon, as some analogous ones were published in *L'Imparziale* of Florence, in the year 1868. Quinine was, however, in these latter instances added to the opium.—*New York Medical Journal.*

The Pathology and Treatment of Scarlet Fever.—Dr. Renfrew, of Glasgow, read a paper on this subject before the British Medical Association. He stated that scarlet fever is one of the zymotic diseases, which diseases are produced by an organized substance entering the body, which has the power of multiplying itself. In multiplying itself the blood is disordered, the nervous system deranged, the circulation quickened, and the secretions and excretions are changed. The poisons of the zymotic disease are not thrown off by the usual eliminating organs, but each poison is eliminated by the particular part of the body—small-pox by the skin, cow-pox at the point of introduction, enteric fever by the lower part of the ileum, scarlet fever by the fauces and nose. When the poisons are thrown off there is always irritation and inflammation. As the poison of scarlet fever is thrown off by the fauces and nose, a large portion must pass into the stomach to be reabsorbed, intensifying and prolonging the disease. The remedies given in scarlet fever should be those that will destroy the poison; moderate and assist physiological changes. To accomplish these ends a mixture of chlorate of potash and tincture of steel is given, which contains chlorine, muriatic acid, iron, and chlorate of potash. The chlorine destroys the poisen ; the acid supplies acid to the blood, which is in a subacid condition; the iron improves the red disks, which are in a black and melanosed condition ; the chlorate of potash supplies oxygen, to assist in oxidizing the disintegrated material that is floating in the blood. —*Lancet.*

The Oxalate of Cerium in Dyspeptic Vomiting.—Dr. S. A. Lucas employs the following prescription in dyspeptic vomiting:

℞—Cerii Oxal. . . . . gr. 36.
Ext. hyosciam, . . . . gr. 24.
Div. in pil. . . . No 12.
Cap. j. ter in die.

This effected a cure, when all other well-known remedies failed. —*Med. Press and Circular.*

Typhoid Fever.—Dr. Q. C. Smith, regarding typhoid fever as strictly a nervous disorder, claims great success in its treatment by the following prescription, after removing complications :

℞—Glycerine, . . . . f. ℥ ii.

Fld. ext. Valerian, . . . f. ʒ iv.

Oil Sassafras, . . . f. ʒ ij. M.

Sig.—A teaspoonful every hour or two hours through the day He has the patient sponged at early bed-time, with mild, tepid soap suds, rinsing off with milk-warm water. For relief of pains of spine, neck, &c. he uses the following liniment :

℞—Chloroformi, . . . f. ℥ i.
Gum. Camphor, . . . ʒ ii. M.

Applied for a short time to painful parts by wetting a thin cloth.—*Med. and Surg. Reporter.*

Atomized Medication for Asthma.—Dr. M. F. Basset, of Quincy, Ill., reports several cases (Med. & Surg. Reporter,) in which this distressing malady was most satisfactorily treated by the inhalation of medicated spray. The cases reported had resisted the usual treatment. He uses the following anti-spasmodic and expectorant medication :

℞—Ext. hyosciami fl'd.

Ext. lobeliæ, fl'd, aa, . . . f. ʒ j.

Aquæ, dist. . . . f. ℥ j. M.

In practice, he recommends the hand atomizer—one throwing a continuous and copious jet of spray.

Neuralgic Liniment.—℞—Lin. chlorofori, lin. belladonna, equal parts. S. Apply topically to the affected part.

Prescription for Whooping-Cough.—Dr. Mockelcan calls attention (in the Dominion Med. Journal,) to the value of *sulphuret of potash* in *whooping-cough.*

| | | |
|---|---|---|
| ℞—Sulphuret of Potash, | . . | grs. xxxii. |
| Simple Syrup, | . . | ℥ i. |
| Distilled water, | . . | ℥ iii. M. |

S. A teaspoonful three times a day, for a child one year old. The dose of the sulphuret should be one grain for each year up to four, and after that, half a grain for each additional year. As the drug easily spoils by keeping, it is important to have it fresh. When the solution is of a greenish color, it is good.

Prescription for Catarrhus Vesicæ.—M. Mallez has found (Lancet Jan 2,) the following solution injected *into* the bladder in *catarrhus vesicæ*, very efficacious :

| | | |
|---|---|---|
| ℞—Iodid. Potassium, | . . . | grs. xv. |
| Tinct. iodidi, | . . . | gutt. lxv. |
| Aquæ. | . . . | ℥ x. |
| ft. solutio. | | |

When the pain is very annoying, add 15 grs. of ext. belladonna to the above. He has also used carbolic acid, nitrate of silver, and hypophosphite of soda, with advantage.

In the Diarrhœa of Infants due to Indigestion,—the following prescription will be found valuable :

| | | |
|---|---|---|
| ℞—Pulv. ipecac, | . . . . | gr. i. |
| Pulv. rhei, | . . . . | gr. ii. |
| Soda bicarbonatis, | . . . | gr. iv.—vij. m. |
| Div. in chart, No. x ij. | | |

S.—One powder every four to six hours to an infant one year old.—*Therapeutical Bulletin.*

Diarrhœa of Children.—Dr. C. F. J. Lehlback says that he has found no combination of remedial agents better in cholera infantum, or in children's diarrhœa, than the following :

| | | |
|---|---|---|
| ℞—Pepsin, | . . . . | ʒ ss. to ʒ i. |
| Bismuth, sub. nit. | . . | ℈ ss. |
| Pulv. opii, | . . . | gr. 1-6 to 1-2. |
| Acid carbol. | . . . | gr. 1-8 to 1 2. |
| Quin. Sulph. | . . . | gr. 1 to ii. M. |
| ft. chart. | . . . | No. x. |

S.—One powder every two to four hours, as the case demands, for a child from one to one and a half years old. In some cases, the opium is omitted, or bromide of potassium given in its place.

Obstinate Vomiting.—Dr. O. C. Alexander reports a case of obstinate vomiting which was immediately arrested by the subcutaneous injection of one-sixth of a grain of morphia.—*Med. and Surg. Reporter.*

Treatment of Asthma.—Dr. D. C. McCampbell, Holly Springs, Miss., derives much benefit from the following method of treating Asthma. Take strips of letter paper, and dip them in a saturated solution of nit. potass.; dry them, and during the paroxysm let the patient ignite one at a time, and inhale the fumes. In a majority of the cases, this will cause the paroxysms to cease. In the interval he uses nitric acid two or three times daily.—*Med. and Surg. Reporter.*

Nux Vomica in Chronic Dysentery.—Dr. De Savignac, who has had large opportunities for observation in the marine hospitals of Toulon, claims excellent results in the use of nux vomica, in dysentery and dysenteric paralysis. His theory is, that the cause of the disease lies in an affection of the spinal cord, which causes paralysis of the motor nerves of the large intestines, and of the vaso-motor nerves which supply its blood-vessels.—*N. Y. Medical Gazette.*

Neuralgia.—M. Bertrand, Paris, has used the following ointment with perfect success, in neuralgia, when other remedies had been tried without effect:

℞—Veratriæ, . . . . grs. v.

Morph. Sulph. . . . grs. iij.

Adipis, . . . ℥ i. M.

Sig.—Rub painful parts with the ointment frequently, when the paroxysms of pain are at their height, and as often as they require. Two or three frictions suffice, in the majority of cases.—*Therapeutical Bulletin.*

Nervous Affections of the Abdominal Organs in Women.—Dr. Dumas, of Montpelier, France, employs the subjoined formula in these affections:

℞—Castorei, . . . . grs. xxx.
Camphoris, . . . . grs. xv.
Pulv. Opii, . . . . gr. viii.
Confectio rosæ, . . . . q. s. M.
ft. pil. No. xv.

S.—Use—*pro re nata.*—*Therapeutical Bulletin.*

Treatment of Nevus.—Dr. Henry Bateman applies a tartarized antimony plaster to the surface of the tumor, allowing it to remain until pustules resembling small-pox are produced. He takes one part of antim. tartarizatum, mixed, with two parts of melted empl. resinæ, and then spread upon leather or linen. It ought to cover the entire nevus. If it causes too much inflammation, poultices, &c., must be applied. Should the skin be unusually unsusceptible, equal parts of the above should be used.—*Lancet.*

Carbolic Acid in Eczema.—Dr. D. S. Reynolds, of Louisville Ky., reports several cases of eczema, occurring on young children, speedily cured by carbolic acid lotion to the parts. The following formula was used in one case :

℞—Acid carbolic cryst. . . grs. xvj.

Olei Olivæ, . . ℥ ii ft. sol.

Sig. Apply night and morning. The diseased surfaces should be well cleansed by warm water without soap, before applying the lotion. Where the bowels are deranged, he directs ten grains of sulphite of soda to be given in water, every two hours.—*Medical and Surgical Reporter.*

Carbolated Alcohol.—Dr. W. H. Williams recommends the following formula for using carbolic acid, to incised wounds, injuries, minor operations, &c.

℞—Acid carbolic, . . . gr. v.

Alcohol dilut. U. S. P. . . f ʒ ii.

Aquæ dist. . . f ℥ i. m.

*Med. and Surg. Rep.*

Iodized Syrup of Horse Radish.—Dr. Petit says in *bronchorrhœa*, when there is considerable secretion, under the influence of which patients soon grow thin and lose all appetite, the use of Grimault's iodized syrup of horse-radish, in daily doses of three or four tablespoonfuls, has a most beneficial effect. From being purulent and muco-purulent as before, it becomes mucus, then decreases in quantity. The appetite soon returns and the excessive perspiration ceases.—*Tribune Medicale.*

Ergot of Rye in Neuralgia.—Dr. Woaks, of London, has employed with satisfactory results, the ergot of rye in various forms of neuralgia. The following is one of his prescriptions :

℞—Potassæ bicarbon., . . . ʒ iss.

Infusi Ergotæ, . . . f. ℥ vj.,

Ext. Ergot Liquid, . . . f. ʒ j. m.

Sig. One ounce every four hours.—*Jour. Cutaneous Med.*

Ergot in Puppura.—Dr. Bauer reports great success in the treatment of puppura hemorrhagica with secale cornutum. He gives eight to ten grains three times, or oftener daily, until hemorrhagic manifestations cease. When anœmia remains, he treats it with chalybeates.

Uterine Hemorrhage.—The oil of erigeron has been found to exert a controlling influence in arresting uterine hemorrhage, given in six drop doses, every fifteen minutes, dissolved in a teaspoonful of alcohol.

PROPHYLAXIS OF SCARLET FEVER AND MEASLES.—Dr. J. C. Peters regards the much vaunted belladonna as a prophylactic in these diseases, as unreliable and dangerous. He now never uses the belladonna for this purpose, but relies entirely on the sweet spirits of nitre. It is a mild and safe remedy. Comparative experiments prove that it is more reliable than belladonna, and far less dangerous.—*N. Y. Med. Gazette.*

SUBSTITUTE FOR DOVER'S POWDER.—Dr. Chapin has not used Dover's powder for twenty years, much preferring his substitute as more palatable and effective; which is as follows:

℞—Opii pulv., . . . . ʒ i.

Ipecac, pulv., . . . . ʒ ss.

Camph. pulv., . . . . ʒ ij.

Saccharum, . . . . ʒ iv.

Mix thoroughly. This contains one grain of opium in eight grains of the powder. He frequently uses morphia, in due proportion, instead; or omits the opium altogether when contra indicated.—*Med. and Surg. Reporter.*

TREATMENT FOR TAPE WORM.—Dr. Wm. M. Turner remarks that the following plan for expelling either the tænia lata or tænia solium, never fails. First: At bed time give the following cathartic:

℞—Pulv. potass. et Rhei, aa . . grs. 10.
Hydr. Chlo. Mit. . . grs. ii. m.
Chart . . . . No. 1.

The next morning, after a *decided catharsis*, and on an empty stomach, give:

℞—Kooso, pulv. . . . . ʒ iv.

Aquæ, . . . . f. ℥ ix. M.

Sig.—Take in a quarter of an hour's time, in three doses. The action is speedy, and the danger none.—*Med. and Surg. Report.*

DR. BRINSMADE'S DIAPHORETIC POWDER.—

℞—Morphia Sulph. . . . . ʒ i.

Camphor.

Cretæ prep.

Saccharum, . . aa . ʒ xx.

Mix intimately. Sig. Dose 10 grains.

STRUMOUS ENLARGEMENT OF THE LYMPHATICS.—Dr. J. Waring-Curran, after trying syrup, iodide of iron, iodide of potassium, quinine, cod-liver oil, without effect in these cases, found the enlarged glands to speedily disappear by giving *Iodide of ammonium* in 3-grain doses twice a day.—*Med. Press and Circular.*

Use of Sarsaparilla in Syphillis.—Dr. T. Clifford Allbut states (Practitioner, May, 1870,) that the anti-syphilitic effects of sarsaparilla depend upon the dose in which it is given, and that given in adequate doses, it is one of our best remedies. It has been used in the Leeds Infirmary for at least a quarter of a century, in the form of decoction, and it is made there in large quantities. "Of this decoction, which differs only in unimportant details from the compound decoction of the Pharmacopœia, we administer from four to ten ounces three times a day, or prescribe some such quantity as a pint or a pint and a half to be taken at will during the twenty-four hours. This medication is expensive, no doubt, but that treatment is the cheapest which most quickly cures the patient. The cases in which Sarsaparilla is most useful are cases in which the system is thoroughly infected with syphillis, during the tertiary and visceral modes of its appearance.

"In persons who are in a thoroughly cachœctic state, who have lost flesh and strength, and who are suffering from sluggish ulcerations and indolent gummata, the sarsaparilla is really of very great value. I believe there is scarcely a practitioner among my readers who will not rejoice to hear of a remedy which will help him to cleanse and to re-establish old syphilitic patients —patients whose constitutions have been undermined by want of nourishment or by excesses, who have gone through many courses of mercury, whose irritable mucous membranes will not bear any more iodide of potassium, and who are so sallow, so worn, so broken down, so eaten up by disease as to seem fit only for the grave. These persons clear up on such quantities of sarsaparilla as I have named, and it is here that the drug fills so important a gap. It need not, and it will not, supersede mercury and iodide of potassium in straightforward cases, but it has its place where these means have failed, or where they are on some grounds to be avoided. How far we are right in claiming this important place for sarsaparilla can only be known after an extended use of the drug according to our method by the profession at large.—*American Journal of the Medical Sciences*, July, 1870.

Treatment of Infantile Erysypelas.—Physicians of Stockholm and Strasburg speak highly of the use of the warm bath in infantile erysipelas. The temperature of the bath should be about 85 deg., and hot water should be added gradually, after the child is immersed, until the temperature is 105 deg. to 110 deg. At the lapse of ten to thirty minutes, according to the age and strength of the child, it is removed and wrapped in a warm cloth for two hours, when it usually falls into a quiet sleep. No medicines are given.

Calendula Officinalis.—Marygold.—Dr. A. Livèzey asserts that a tincture of the flowers of Marygold is vastly superior to that of arnaca. He has used it for many years to all kinds of wounds, by saturating a piece of lint, and applying it to the part.

Sulphate of Manganese in Chorea.—Dr. Hammond mentions two cases of Chorea treated successfully by him with this remedy, after the failure of other medicines. One girl, fourteen years old, took 5 grains three times a day; the other, a boy of 15 used the iodized cod-liver in addition.—*N. Y. Med. Gazette.*

Vermifuge.—Santonin and podophyllin in the proportions of 8 to 1, is a convenient and admirable *worm medicine.* One or two grains, *ter die*, the dose.

Subnitrate of Bismuth in Diarrhœa of Young Children.—Heller, in the Deutsches Archiv fur Klinische Medicin, vol vi., recommends, in the treatment of the diarrhœa of early life, the subnitrate of bismuth, to the extent of from thirty to sixty grains. In the commencement, Dr. H. gives a dose of the remedy every hour until the diarrhœa is arrested, which usually occurs at the end of twenty-four hours. He has never seen any bad result from the use of the remedy. During the continuance of the diarrhœa the patient is to be debarred the use of milk.—*D. F, C. in the American Journal of Medical Sciences.—Nashville Journal of Medicine and Surgery.*

Sulphate of Nickel in Neuralgia.—A case of obstinate neuralgia is related which was cured by sulphate of Nickel, in doses of half a grain, three times a day. At the end of a week a dose of one grain was given. Its sedative action was speedily manifested in reducing the pulse and procuring sleep. All symptoms of the paroxysm disappeared. The remedy seems worth a trial.—*Physician and Pharmaceutist.—Oregon Medical and Surgical Reporter*, April, 1870.

The Use of Pepsin.—Dr. E. P. Hurd ridicules the idea that pepsin can be of the value as an aid to digestion that many seem to regard it. Most of the substance of the pepsin we use is *starch*, and in any case, be it never so strong, a scruple of it can only digest eighty or ninety grains of aliment.

Boudault's pepsin only digests about four times its own weight of albumen.

How far is our ordinary dose going to help a poor victim of indigestion through the solution of an ordinary meal?

He strongly suspects that with regard to the real effect of this agent we are very much deceived.—*Medical and Surgical Journal.*

INDUCTION OF PREMATURE LABOR.—Dr. Barnes has successfully induced premature labor for some years in the following manner: First, overnight, pass an elastic bougie six or seven inches into the uterus, coil up the remainder of the instrument in the vagina; this will keep it *in situ.* Next morning some uterine action will have set in. In the afternoon, at an appointed time, proceed to accelerative measures. Before rupturing the membranes, adapt a binder to the abdomen, and let this be tightened, so as to keep the head in close apposition to the cervix. This prevents the cord from being washed down. Dilate the cervix by the medium or large bag, until the cervix will admit three or four fingers. Then rupture the membranes, and before all the liquor amonii has escaped, introduce the dilator again, and expand until the uterus is open for the passage of the child. If the presentation is natural, and the pelvis good, leave the case to nature. If not, accelerate with instruments. Twenty-four hours, in all, should see the completion of the labor.

VALUABLE EMMENAGOGUE.—Dr. J. M. Da Costa uses *apiol* in 4-grain doses, in the form of a granule or "pearl" four times a day, in amenorrhœa, when there is no uterine disease, to be taken three days before the expected period. Dr. Tilt says it acts like a charm, given every two hours in dysmenorrhœa, as soon as the pains begin, but is only useful in cases where there is no uterine disease.

TREATMENT FOR INGROWING TOE NAIL.—Dr. J. M. Quigley has, in every case, had unfailing success by the following plan of treating ingrowing toe-nails: A triangular notch is made midway in the free edge of the nail extending to its body (the piece out of which should be in size about like the tooth of a tenon-saw;) from the pointed margin of this notch, a furrow is made, as near to the quick as possible, without penetrating it, through the middle of the root as far as the duplication of the skin; a piece of cork is then inserted under the nail, whose bulk is large enough to extend a few lines on either side of the notch, as well as to completely fill, without uneasiness, the inter-space between the skin and extremity.—*Med. & Surg. Reporter.*

STIMULATING EMMENAGOGUE.—Dr. T. Hawkes Tanner employs the following formula:

| | |
|---|---|
| ℞—Potassi bromidi, . . . | ʒ i. |
| Tinct. Cantharidis, . . . | f. ʒ iss. |
| Tinct. Cinnamoni, . . . | f. ʒ vj. |
| Aquæ q. s. ad. . . . | f ℥ iss. M. |

Sig. Desert Spoonful three times a day.

*Therapeutic Bulletin*, compiled by Dr. Geo. H. Napheys, M. D., Philadelphia.

Belladonna in Arresting the Lacteal Secretion.—Dr. D. W. Stormont, of Topeka, Kansas, (Leavenworth Med. Herald,) mentions two cases of mamary abscess, in both of which the secretion of milk was stopped by the application of belladonna (ext. belladonna ʒ ii., aquæ f. ℥ i.) painted over the breast. The lacteal secretion may be restrained, or entirely dried up, at the option of the physician, in one breast, without producing much effect in the other. Hence he considers it invaluable in mammary abscess, both as a prophylactic and as a curative agent. The patient should be cautioned against nursing the child from the breast to which the belladonna has been applied.

Calabar Bean in Tetanus.—Calabar bean has been used quite beneficially in tetanus. Indeed the array of testimony in its favor in this fearful malady, seems to be conclusive as to its great value. Among others, we call attention to Drs. Bostin & Curren's (Chicago Med. Jour.) case, treated with the Calabar bean and morphia. For a portion of the time a grain and a half of morphia, and three grains of powdered bean in glycerine, were given every hour—quieting and relieving the patient. The patient was restored.

Chloride of Sodium in Dysentery.—Dr. L. Woodruff has found the following prescription valuable in Sporadic and epidemic dysentery :

℞—Morph. Sulph. . . gr. i. to iss.
Sodii Chloridum, . . ℈j. m.
ft. chart. No. vi—S. One every 4 hours.

He premises the treatment by calomel and opium, followed by Castor Oil. He regards this treatment superior to any he has ever employed.—*Med. & Surg. Reporter.*

Ascarides.—Dr. L. B. Balliet regards Santonine as the best remedy for Ascarides. He gives it in doses of 5 to 10 grains, morning and evening, or every evening only. He does not combine it with any cathartic, as it is apt, when thus combined, to produce vomiting.—*Med. & Surg. Reporter.*

Lobelin for Rigid Os Uteri.—Dr. Kilner, of Sullivan, Ill., strongly recommends the *lobelin*, the active principle of *lobelia inflata*, for dilating the os uteri and perineum during labor. He uses it in the form of a rectal suppository, five grains, rubbed up with cocoa butter, q. s., and he states that fifteen to twenty minutes will usually suffice to bring the desired effect. He thinks no danger need be apprehended in its use. While we can imagine its great relaxing properties, yet would recommend trial of other expedients before resorting to it.

Carbolic Acid in Rubeola.—Dr. T. J. Williamson has found much advantage from the use of Carbolic Acid in malignant form of measles. In one case his prescription was as follows:

℞—Syrp. Scillæ, c. . . . . f. ℥ i.

Syrp. ipecac,

Tinct. lobelia inf. āā . . . f. ʒ i.

Carbolic Acid, . . . . gtt. xvj. M.

S. Teaspoonful every hour.

And as a wash for mouth—

℞—Carbolic Acid, . . . . ℈ ii.

Chlo. Potass. . . . . ℈ i.

Mellis desp. . . . . ℥ ss.

Aq. Camph. . . . . f. ℥ ii. M.

S. Gargle the mouth every three or four hours.

[*Richmond and Louisville Journal.*

Sulphurous Acid in Pyrosis.—In every instance, Dr. Lawson asserts (in the Practitioner,) in which *Sulphurous Acid* has been employed, it has, in a very short time, given for *pyrosis*, completely arrested the water-brash secretion. The doses of the acid (B. P.) vary from m xxx to ʒ i, three times a day, shortly before meals. Bitter infusions may be employed, as a vehicle, but plain distilled water is best.

Digitalis in Menorrhagia.—Dr. J. P. Chesney reports a number of interesting cases of Menorrhagia cured by digitalis after other remedies had failed. He thinks it has a tonic action in cases of relaxation and debility of the tissues; and that it will be found useful in leucorrhœa, spermatorrhœa, gleet, diarrhœas, &c. —*Leavenworth Med. Jour.*

Simple Method of Arresting Obstinate Epistaxis.—A writer in the Gazette des Hopitaux states that attentive observations of the face of a patient attacked with epistaxis will detect a slight intermittent movement of the soft parts near the ala of the nose on the side where the blood is flowing; even if this pulsation be not seen, it may be felt. Pressure with the finger over this branch of the facial artery is said to arrest the hemorrhage immediately.—*Medical Gazette.*

Belladonna in Infantile Icterus.—Dr. Waring-Curran (Medical Press and Circular,) gives *tinct. belladonna* in two-drop doses in *infantile icterus*, under the idea that the bile is not *suppressed* but simply *retained*, by the contraction of the *ductus communis choledochus.* The treatment is successful—the child becomes quiet, falls asleep and passes bile freely.

Bromide of Potassium for Infants.—M. Moulard-Martin, says the Practitioner, is repeatedly called to see infants in other respects healthy, who, during the early months of their existence, cannot rest, and who wear out, in consequence of loss of sleep, those who are about them ; there are other children who sleep during the day, but never at night. In these cases, when tepid baths, infusion of poppies, orange-flower water, &c., have failed, bromide of potassium succeeds in a remarkable manner. It should be given in moderate doses—from five to twenty centigrammes is perfectly tolerated by young children. By its sedative action it cures insomnia.

Citric Acid in After-Pains.—Dr. J. B. Chagnon (Canada Med. Jour. May 1870,) states that *citric acid* has never failed in his hands in "after pains." Five grains of the acid are given in two or three ounces of water every five hours.

Hypocondriasis.—In cases of hypocondriasis, with tendency to Melancholia, very small doses of Strychnia will, in many instances, give unmistakable relief.

Tonics in Chronic Bronchitis and Emphysema.—Dr. J. C. Thorowgood remarks that tonics, however they are feared by patients suffering from these diseases, are of great use, especially iron and nux vomica, or strychnine in small doses. The presence of expectoration and of occasional fits of severe nocturnal asthma is no drawback to their administration ; and not unfrequently the nux vomica will relieve these attacks most effectually. Arsenic, at times, will act like a specific in improving the breathing—it appears to promote nutrition, and is of great service.—*Practitioner.*

Cholera.—Dr. Bates, of Manchester, observes, whatever the origin of cholera may be, it produces "clonic" spasms of the voluntary muscles. This suggested to him the subcutaneous injection of morphia in a case fully in the stage of collapse. On returning to see the effect in an hour afterwards, he "found the abdomen warm, which had previously been cold." He then repeated the injection, and in an hour afterwards found reaction becoming established. This affords a valuable hint as to the treatment of sporadic cholera.—*Synopsis, Braithwates Retro.*

Permanganate of Potassium in Rheumatism.—Dr. W. S. King reports four cases of rheumatism relieved by the following prescription :

℞—Potass. permanganitis, . . gr. ii.
Syrp. Sarsaparilla . . f ℥i. m.
S.—One teaspoonful three times a day.

[*Med. & Surg. Reporter.*

REMEDY FOR WHOOPING-COUGH.—An experienced practitioner in an article published in the *Canada Med. Journal*, says that the following prescription for whooping-cough is the best he has met with:

| | | |
|---|---|---|
| ℞—Ammon. bromide, | . . . | ʒ i. |
| Acid hydrocyan dil., | . . gutt. | xx. |
| Tinct. sem. Stramonii, | . . gutt. | xx. |
| Water and Syrup, | . . . | ℥ iv. M. |

S—A teaspoonful three times daily to a child two years old. It often relieves in twenty-four hours. Two or three grains of the bromide of ammonium may be given three times daily.—*Medical Record.*

SUPPRESSED BRONCHIAL SECRETION.—Dr. Labat, Paris, gives acetate potash in these cases. To a child 18 months old, he gave acetate potass, grammes 8; water grammes, 120—in teaspoonful doses every half hour. He is convinced this medicine is capable of producing abundant mucus expectoration.

LOCAL PARALYSIS.—Dr. Brown—Sequard uses the following formula in local paralysis:

| | | |
|---|---|---|
| ℞—Strychnæ Sulph. | . . . . | grs. ij. |
| Chloroform, | . . . . | ℥ i. M. |

Apply half, night and morning, by brisk friction to the part.

TREATMENT FOR CHOREA.—Dr. Rodolphi (Gazetta Med. Lombard,) having had ample opportunity of treating *chorea* at the Brescia Hospital, and trying a great variety of remedies, asserts that the *muriate of lime* is the best. He proceeds to its employment by a purgative, composed of castor oil, calomel and santonine. When no cerebral hyperæmia is present, he then begins with the muriate, giving fifteen grains in the twenty-four hours. Improvement commences at once, and the cure is completed in one or two weeks. An addition of 7 centigrammes *per diem* of extract of belladonna augments the efficacy of the muriate.

APPLICATION OF ICE TO THE SPINE IN EXCESSIVE MENSTRUATION.—Dr. Wm. C. Cooks reports several cases where ice, applied to the lower portion of the spine, appears to have produced the happiest effects. Either Chapman's spinal bag, or a bladder may be used. The ice must be broken in small fragments, placed in the bag until two-thirds full, and placed under the patient lying on her back, immediately to the lower portion of the spine. It should be retained to the part from half an hour to two hours.—*Med. & Surg. Reporter.*

